# Reply to: “Enhancing biliary protection via HOPE: Beyond bile composition to cholangiocyte resilience”

**DOI:** 10.1016/j.jhepr.2026.101930

**Published:** 2026-06-17

**Authors:** Frederik Schliephake, Isabella Lurje, Georg Lurje

**Affiliations:** 1 Department of General Surgery, Division of Transplantation, Medical University of Vienna, General Hospital of Vienna, Vienna, Austria; 2 Department of Surgery, Charité – Universitätsmedizin Berlin, 13353 Berlin, Germany; 3 Department of Hepatology and Gastroenterology, Campus Virchow-Klinikum and Campus Charité Mitte, Charité – Universitätsmedizin Berlin, Germany

To the Editor:

We thank Wu *et al.* for their commentary on our translational study investigating the effects of hypothermic oxygenated machine perfusion (HOPE) on bile composition after liver transplantation and welcome the opportunity to address these points in detail. Our study harnessed liver and postoperative bile samples collected in a randomized controlled trial (RCT) comparing HOPE and static cold storage (SCS).^1^ We demonstrated that HOPE limited intrahepatic bile acid accumulation during allograft reperfusion, and resulted in improved biliary lipid secretion, reduced biliary bile acid-to-phosphatidylcholine ratio, and accelerated the normalization of serum bile acid levels after transplantation.^2^

The authors correctly identify a discrepancy between the significant improvement in biliary lipid secretion observed in HOPE-treated grafts and the absence of a statistically significant difference in biliary stricture rates. Here, we would like to emphasize that the investigated mechanism of bile acid toxicity has been specifically proposed to play a role in the pathogenesis of non-anastomotic strictures (NAS)^3^ which have a low incidence in the investigated cohort of donation after brain death (DBD) recipients, compared to donation after circulatory death (DCD) cohorts, limiting the statistical power to detect differences in biliary complication rates in our 26-patient cohort. Validation of the bile analysis in a dedicated DCD cohort, where the incidence of NAS exceeds 25% after conventional SCS,^4^ and confers substantial posttransplant morbidity, is an essential next step. HOPE significantly reduced the incidence of NAS in an RCT performed in DCD organs, with long-term data demonstrating around a 50% reduction in long-term biliary strictures.^4^

Wu *et al.* emphasize deep peribiliary glands (dPBG) and the peribiliary vascular plexus (PVP) as critical determinants of biliary regeneration, and we share their view that these structures play an important role in ischaemic cholangiopathy. In this context, we believe the pathogenesis of ischaemic cholangiopathy can be framed as a two-hit injury to the dPBG niche (Fig. 1). In the first hit, donor warm ischaemia causes ATP depletion and mitochondrial injury, leading to PVP arteriolonecrosis.^5^ Loss of the PVP reduces blood supply to the dPBG, placing progenitor cells in a state of hypoxia that impairs their proliferation and differentiation, as confirmed histologically in NAS of patients requiring retransplantation.^6^ In the second hit, ischaemia-reperfusion injury impairs the function of the transporters ABCB4 and ABCB11, resulting in a window in which bile salt secretion recovers more rapidly than phospholipid secretion.^7^ The resulting high free-bile-acid concentration in the biliary lumen, combined with disruption of the bicarbonate umbrella, allows hydrophobic species to penetrate the periductal submucosa. Periductal bile acid exposure at this anatomical level has been shown in a mouse extrahepatic bile duct explant model to cause cholangiocyte monolayer disruption, increased submucosal collagen deposition, and myofibroblast activation.^8^

**Fig. 1:**
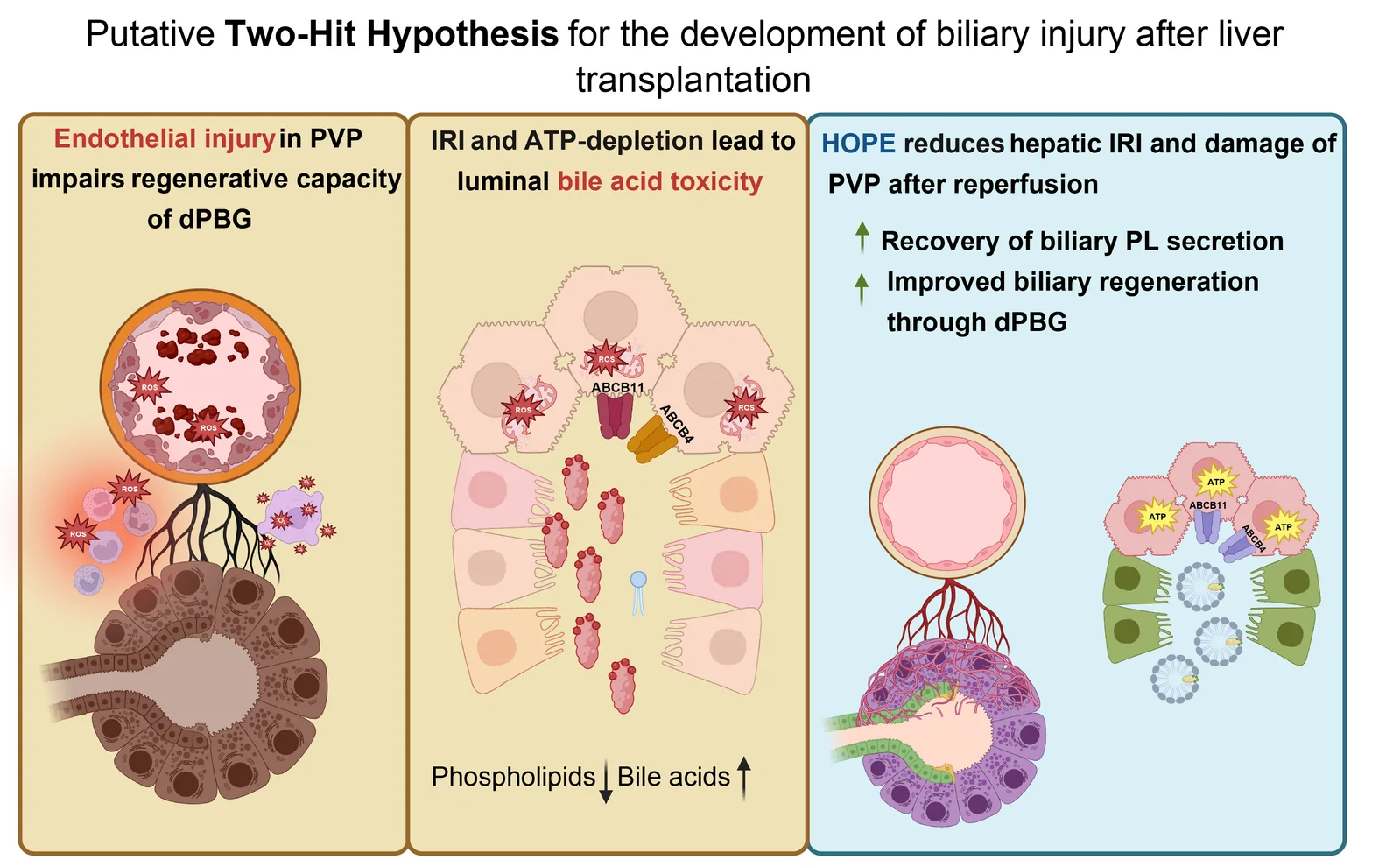
**Proposed “two-hit” mechanism linking ischemia–reperfusion injury, bile composition, and biliary damage.** dPBG, deep peribiliary glands; HOPE, hypothermic oxygenated machine perfusion; IRI, ischemia–reperfusion injury; PL, Phospholipids; PVP, peribiliary vascular plexus; ROS, reactive oxygen species.

HOPE may address both hits simultaneously. By restoring mitochondrial ATP synthesis before reperfusion, HOPE reduces the reactive oxygen species burst that drives PVP endothelial injury^5^ and restores biliary phospholipid secretion more rapidly after reperfusion,^2^ likely reducing the free-bile-acid window that confers periductal toxicity. Van Rijn *et al.* showed significantly less injury and cell loss in both periluminal and deep PBG after reperfusion in DHOPE-treated DCD grafts compared to controls.^9^ We acknowledge, however, that direct evidence linking a high biliary bile salt-to-phospholipid ratio to bile acid-induced apoptosis and progenitor cell loss within the dPBG niche is currently lacking.

The authors propose investigating the synergy between end-ischaemic HOPE and postoperative ursodeoxycholic acid administration. Post-transplant ursodeoxycholic acid supplementation was part of our centre’s standard protocol for all recipients; thus, this treatment effect was already present across the cohort.

The authors propose to utilize organ-on-a-chip models to study bile acid toxicity in cholangiocytes. A complementary and physiologically more complete platform to study biliary regeneration is provided by long-term *ex situ* normothermic machine perfusion. This approach preserves the intact three-dimensional architecture of the biliary tree and permits serial sampling of bile, perfusate, and tissue over extended periods.

Recently, long-term normothermic machine perfusion was applied to discarded donor livers to reproduce biliary regeneration *ex situ*.^10^ This enabled the identification of key exocrine mediators of this process including IL-6 and VEGF-A, both known regulators of progenitor cell activation in the dPBG niche.^10^

In summary, our study identifies altered biliary composition as a distinct feature of HOPE-treated grafts. Future studies in DCD donation and advanced *ex situ* models will be instrumental in defining how alterations in bile composition interact with biliary regeneration to prevent the development of ischaemic cholangiopathy.

## Authors’ contributions

F.S. conceptualization, writing I.L.: writing – review and editing G.L: conceptualization, writing – review and editing.

## Financial support

This work was funded by the 10.13039/501100001659Deutsche Forschungsgemeinschaft (LU 2185/2-1).

## Conflicts of interest

FS reports receiving speakers’ fees from XVIVO; GL reports receiving research funding and speakers’ fees from Astellas Pharma, XVIVO, Bridge to Life, Organ recovery systems, Wyss Liver4Life, Orphalan and Aferetica S.R.L, and is on the advisory board of OrganOx, outside the submitted work.

Please refer to the accompanying ICMJE disclosure forms for further details.
